# The Advance Care Compass– A New Mechanics for Digitally Transforming Advance Directives

**DOI:** 10.3389/fdgth.2021.753747

**Published:** 2021-10-15

**Authors:** Nikola Biller-Andorno, Armin Biller

**Affiliations:** ^1^Faculty of Medicine, Institute of Biomedical Ethics and History of Medicine, University of Zurich, Zürich, Switzerland; ^2^Multi-Dimensional Medical Information Lab, Department of Neuroradiology, University of Heidelberg, Heidelberg, Germany

**Keywords:** advance care planning (ACP), ethics, digital health adoption, patient preferences, patient-centered care, health care improvement

## Abstract

Advance directives allow people to declare their treatment preferences for a potential future state of incompetency. Covid-19, with its high numbers of quickly deteriorating patients requiring intensive care, has acutely demonstrated how helpful it would be for clinicians to have reliable, readily available, up-to-date information at hand to be able to act in accordance with what the individual patient would have wanted. Yet for the past few decades advance directives have fallen short of their potential, for various reasons. At worst, advance directives are perceived as unwieldy legal documents that put excessive demands on patients without providing useful guidance for better care. Recent efforts such as advance care planning have tried to remedy some of these shortcomings but have so far met with limited success. We suggest a new concept—the Advance Care Compass—that harnesses the potential of digitalization in healthcare to overcome many of difficulties encountered so far.

## Advance Directives: Great Aspiration But Difficult To Implement

Advance healthcare directives (ADs) can be considered a key achievement of 20th century Western bioethics and an expression of its core value, autonomy. With technological development in clinical medicine progressing rapidly in the 1960s and 70s, people worried they would die packed with tubes and surrounded by blinking lights, with no escape.

These worries were fueled by landmark cases such as those of Karen Quinlan, Nancy Cruzan or, more recently, Terry Schiavo, in which relatives fought for the termination of life-sustaining treatment they considered to be in conflict with the comatose patients' wishes (1, 2). The US Patient Self-Determination Act of 1990 required US healthcare institutions to inform patients about their right to make decisions concerning their medical care, to refuse treatment and to formulate ADs. Other countries such as the UK (Mental Capacity Act, 2005, Sections 24–26), Germany (German Civil Code, § 1901a, since 2009) or Switzerland have subsequently also provided legal frameworks for ADs (Swiss Civil Code, Art. 370-373, since 2013).

Challenges were identified early on, such as the limited knowledge and readiness of patients to engage with possible future states of severe illness (3). On the other hand, the benefits—particularly an increased chance of offering care that matches with a patient's values and preferences—become ever more important in modern medicine with its multiple treatment options and its recognition of personalization and patient-centeredness as hallmarks of clinical excellence. It comes as no surprise, therefore, that ADs, albeit with certain variations, have been adopted as a legal instrument in many countries around the globe (4).

Yet, the effectiveness of ADs and their actual impact on medical decisions has remained limited, due to a range of factors including unavailability, lack of specificity, or the inability of proxies to accurately express patient wishes (5). It is not surprising, therefore, that numbers have remained relatively low over time: Only about a third of US adults has completed an AD (6), and numbers seem to be largely similar in other countries (7, 8). Although physicians generally recognize ADs and their legal status, they, too, remain somewhat skeptical (9, 10). Only very recently, with the Covid-19 pandemic spread, an increase in completed advance directives has been observed (11).

The emphasis on the legal character of the instrument—rather than its communicative or ethical dimension—does not necessarily make things easier: In Germany, for instance, the Federal Court of Justice ruled that ADs were only legally binding if they were sufficiently concrete with a view to treatment measures or situations (12). This renders the task of completing a living will even more taxing, particularly for people who have little knowledge or experience regarding the treatment choices they wish to determine in advance.

Various strategies have been pursued in order to overcome this dead end:

One response could be to offer standardized AD text modules with concise wording that conforms to legal standards; however, it remains unclear if the modules selected by users genuinely reflect their values and priorities. This concern is particularly relevant for users who do not have the medical and legal background knowledge that is required to make informed choices. Such standard forms are now increasingly available online, behind a paywall or provided for free, sometimes by health insurance companies, raising issues about further use of data. Other patient-directed online formats focus less on producing legally binding living wills but on sharing medical care wishes and preferences that can guide conversations and future decisions (e.g., https://www.ourcarewishes.org). In general, forms, particularly if online-based, are easily accessible and updated; scalability is not an issue.

Another response has been to use the AD mainly as a medical power of attorney, nominating a healthcare proxy who is supposed to make decisions on behalf of the incapacitated patient rather than trying to provide detailed preferences about future treatments that may be difficult to anticipate. However, as studies have shown, proxies are often overburdened with this task and find it hard to distinguish between their own and the patient's preferences (13–15). Not only do they need to be able to identify what the patient would have wanted in a given situation, they need to be able to communicate well in a clinical setting and they have to be available. The quality of proxy decision-making is therefore highly variable.

Physician Orders for Life-Sustaining Treatment (POLST) aim to tackle the challenge that ADs are not always heeded. The idea is that portable medical orders that are based on a conversation between patient and health care professional will be better able to ensure that patients' treatment wishes at the end of life are indeed honored (16). POLST is for frail or seriously ill individuals who are already in medical care (cf. https://polst.org). As POLST is a medical order form and not an advance directive, modifications cannot be carried out by patients themselves but need to involve a licensed healthcare provider (https://polst.org/wp-content/uploads/2020/06/2020.06.05-Patient-POLST-Form-Guide.pdf). This renders POLST less flexible and more resource-intense than AD forms, which do not necessarily have to be signed by a physician; on the other hand, users profit from expert advice and the authority that comes with a medical order.

Finally, Advance Care Planning (ACP) tries to guide patients through treatment options in a structured dialogue with a trained professional (17). Problems of this approach regard upscaling—there is a limited number of health professionals with ACP training, and physicians hesitate to fully engage unless they are reimbursed, which is not the case everywhere (18). Seeking help each time the document is to be updated is quite resource-consuming; it is possible that updates do not happen for lack of resources or because people hesitate to bother the ACP professional again. In addition, not everyone is keen to discuss their private thoughts about health care choices with a third party. Similar to POLST, ACP is mainly for elderly or severely ill patients but less for healthy adults, who nevertheless might profit from an AD (19).

In summary, over the past three decades, ADs have become an established but not uncontested legal instrument that many people feel compelled to use in order to specify what actions shall be taken with regard to their health if they are no longer able to make decisions for themselves. Various initiatives have tried to address core challenges, from the basic epistemic problem of how well we can anticipate our preferences regarding future health states, to implementation issues and the largely open question about impact on care (20–22). So far, all approaches come with important limitations: Whereas an AD form can offer precise, legally valid formulations, requires little resources and is easily updated, it is hard to know if the documented preferences rest on well-informed and well-reflected choices. Proxy decision-makers differ in their availability and may be more or less able to fulfill their role. POLST and ACP provide professional support that may help patients reach decisions that correspond with their wishes and increase clinical impact but are resource intense and limited to frail or severely ill patients who are already in medical care. Outside a clinical care setting where health care professionals are available to discuss and rediscuss care preferences, updates may be difficult.

## Going Digital

Surprisingly enough, digitalisation has so far not been fully harnessed to create innovative solutions that help translate the potential of ADs into real-life benefit for patients and providers. Simply putting forms online does not go far in addressing the multiple challenges ADs and end-of-life choices encounter (23). Pioneering initiatives have started combining preparatory patient-directed online programs with subsequent ACP, which has led to increased active patient participation in ACP discussions and more frequent ACP documentation (24). As this model is coupled to ACP it provides precious support for frail or sick individuals but is less suitable for healthy citizens who want to define their health care wishes in an advance directive.

**Figure 1:**
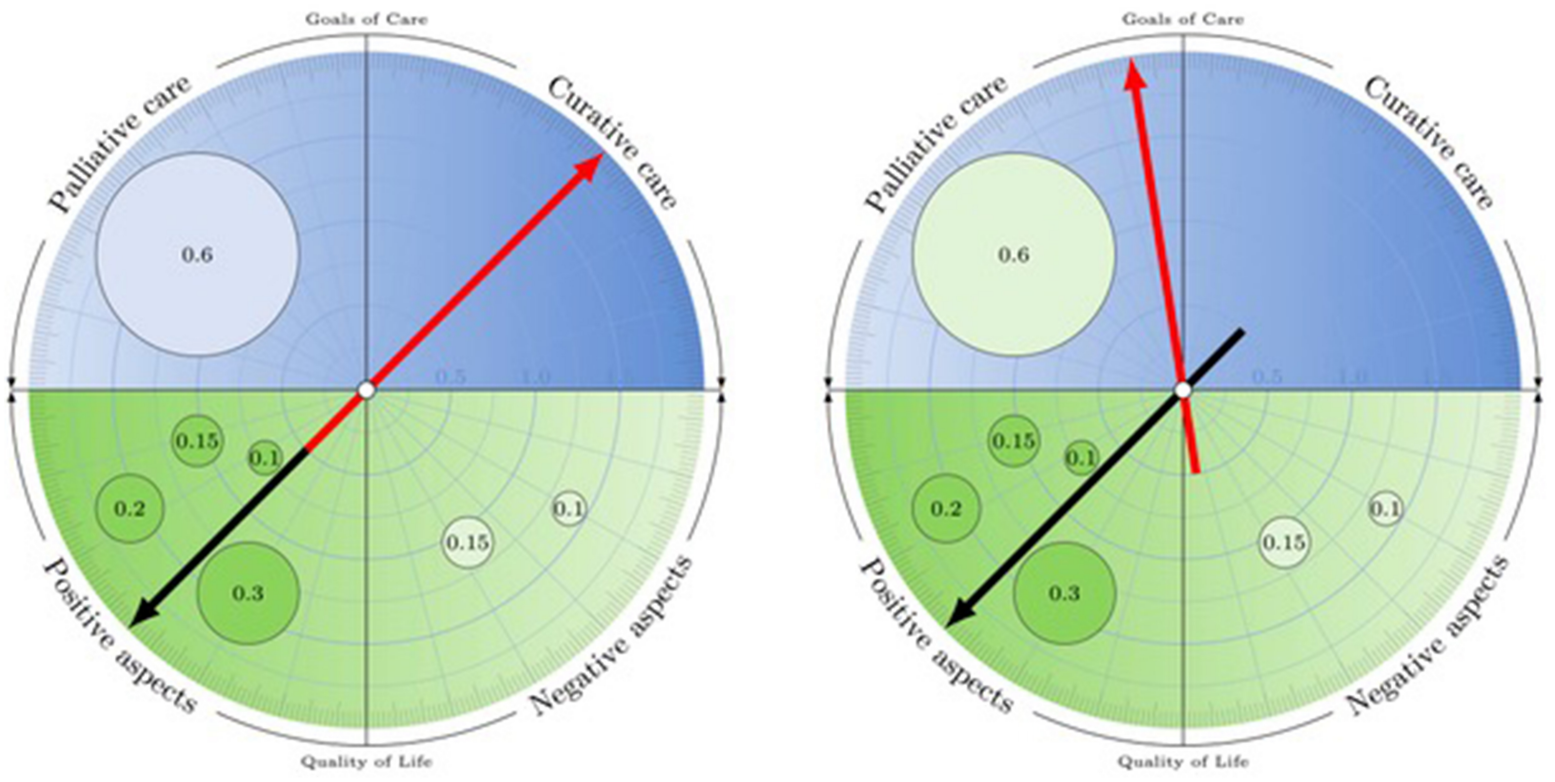
The Advance Care Compass: Simulating the impact of pre-defined events on goal of care preferences. Users are interactively guided through questions regarding (i) their current quality of life (lower half of compass) and (ii) overall goal of care orientation (curative or palliative) in a potential future state of decision-making incapacity (upper half of compass). They then proceed to (iii) more specific questions about how conditions (such as persistent vegetative state) might impact their goal of care orientation (as identified in step ii) and the measures that should be taken or withheld. Users weight their input to tasks (i)–(iii), indicating the relative importance of aspects leading to their decisions.

We set out to explore from an interdisciplinary perspective involving ethics, clinical care and data science how ADs might be digitally enhanced in order to add value to our health care systems, promote patient orientation and engagement, and invite continuous improvement and innovation. Building on the insights and achievements of previous efforts described above and aware of the shortcomings of existing solutions, we invented the concept of the Advance Care Compass (ACC), a mechanics that allows us to transform analog advance care decisions into an innovative digital format (Figure 1). It is important to note that this concept is not considered as an alternative but as complementary to more comprehensive advance care planning processes including human interactions with proxies and healthcare professionals.

The ACC invites users to quantify the positive and negative aspects of their lives with relative weights, leading to a numeric assessment of their current quality of life. This figure is hypothetically translated into a goal of care orientation—palliative for individuals with a negative quality of life, and curative for those with a positive quality of life. The underlying prima facie assumption is that individuals with a bad quality of life will want to invest less (e.g., in terms of burden of aggressive treatments) in the continuation of their lives than people who are very happy. However, someone who is currently badly off might be very willing to receive curative treatment (e.g., to resolve an acute health crisis), and someone who is approaching the end of a rich, fulfilled life might not be interested in invasive medical interventions in spite of a very decent current quality of life. Therefore, the system actively prompts the individual user to explicitly confirm or to adjust the preliminary assessment.

Although the ACC distinguishes two goal of care *orientations*, the underlying conceptual assumption is not that curative and palliative approaches are mutually exclusive but that there is a continuum with shifting priorities—from a clear focus on curation through intermediary stages to a clear focus on palliation. This is graphically represented by the position of the arrow (cf. Figure 1), which may be more toward the middle—indicating an openness to combine approaches (in a way that can subsequently be specified by the user)—or toward the side of palliative or curative approaches only.

If the user has declared to want to receive curative care, he or she is invited to specify what events (if any) would change his or her goal of care orientation toward palliative care, and how much weight the respective event would have. The occurrence of these events and their impact on the goal of care orientation can be simulated individually or cumulatively (cf. Figure 1). Beyond individual simulations of future health conditions, the Advance Care Compass with its quantitative approach makes the model amenable to intra- and interpersonal comparisons and AI-based predictions, opening a new space for future research and development.

The innovative mechanics of the ACC could easily be implemented as an app. In the following, we outline how such a Digital Interactive Advance Directive (d-AD) could harness the potential of digitalization in a way that responds to the challenges advance directives and similar concepts typically encounter (Table 1).

**Table 1:** Key requirements of AD/ACP support tools.

**Challenges**	**Requirements**	**d-AD**
Clear and concise presentation of preferences?	Easy-to-capture core content (“at a glance”)	- Compass format (“Advance Care Compass”) - Dashboard view for quick orientation
Impact on patient-centered/goal-concordant care?	Clearly categorized, quantifiable output	- Vector-based presentation of goals of care facilitates quantitative advance decisions research to establish impact on care as well as inter-individual comparisons and AI-based predictions
Well-considered choice?	Interactive features	- Iterative reflective process through consistency checks - Simulation mode (for patients, proxies and providers) - Active engagement through automated suggestions
Available when needed?	Digital format	- Availability 24/7 (with special emergency data set accessible e.g. through QR or bar code) - Continuous and easy update (with automatic reminders) - Easy scalability, limited resource requirements
User requirements (e.g. health and legal literacy)?	Maneuverability	- Adaptable level of content specificity (overall goals of care vs. concrete measures) - Multimedia guidance (text, voice, videos) and appealing presentation (compass) - Multimodal input (keyboard, speech, digital pen etc.) and output (e.g. in a graphic, classic text-based or text-to-speech format
Compatibility with other clinical systems and services?	Interoperability	- Export and share functions (e.g. invite proxy to digitally confirm role) - Interface with electronic record, AD registers etc. - Integration with expert consultations, e.g. ACP professionals, GPs (inform/discuss, sign, confirm patient legal competency) or lawyer (counsel, compliance with legal standards)

Beyond the obvious advantages of a digital format regarding availability and scalability, the ACC allows the d-AD to visually summarize the main information in a novel, succinct way. This special compass format is a key advantage as clinicians often find it quite impossible to read and interpret many pages of often unclear, inconsistent or medically unreasonable text.

In contrast to standard forms that need to be sequentially filled out, a d-AD provides an interactive, user-centric design. Users can determine the degree and areas of specification, without feeling prompted to declare preferences they may not be certain about, just to ensure the form is complete. Rather, users can concentrate on the messages they do want to convey to their future treatment teams without getting distracted by having to answer sets of standard questions at a level of detail they may consider inappropriate. Formulations are offered and explained that fulfill legal requirements, but adaptations and free text can be chosen as well.

In order to produce robust, well-considered choices, the d-AD offers suggestions based on previous user input, consistency checks and feedback loops, inviting reflection and doublechecking of choices. When the system makes suggestions based on user entries—a statement summarizing overall quality of life (positive or negative), resulting from individual entries, and the general goal of care orientation (curative or palliative), based on current quality of life—it explicitly asks for the individual user's confirmation and explains how adjustments can be made to ensure that the content displayed matches the user's ideas and intentions. The simulation mode helps patients ensure their AD matches their actual care preferences: It allows proxies and health care providers to simulate the effect on goals of care preferences should the anticipated health conditions become a reality.

Implemented as an app, the d-AD concept allows for sharing decisions: The user can identify a legal proxy and a physician (e.g., the GP) who can be contacted for further information. Both can access and sign off on the AD through an electronic interface. The d-AD is thus compatible with a durable power of attorney model, a POLST system, which could easily be integrated into the app, or ACP processes, e.g., with the patient's GP or some other trained professional. This allows users to either fill out their AD independently, to get expert confirmation or to develop the content together with a professional or proxy, as they prefer.

Let's imagine Ms X, a physically and mentally fit 82-year-old lady who was previously a teacher, proud of her computer skills and her affinity to technological innovation. She lives in a senior residency in which she feels well-taken care of. She does not have children or any close relatives. There are many sources of joy in her life, and a few grievances. Overall, the positive aspects clearly dominate. In case her health deteriorated and she lost her decision-making capacity, she would like to receive curative treatment. However, she would not want to be resuscitated. There is one big worry she has regarding her future health: If she fell seriously ill due to a SARS-CoV-2 infection, she would want to go into palliative care immediately. She has two friends who died of a protracted, lonely death in an intensive care unit, and she would not want that for herself.

Further, Ms X is concerned about contracting dementia, a condition that scares her, but her treatment choices would very much depend on the specific details and circumstances that eventuated. Ms X takes time to outline them carefully in the system, specifying milestones in the disease progression that would push her in the direction of palliative care. This task is facilitated by default milestones that the system offers but that can be disregarded or modified. Also, the system provides explanations of medical and legal terms while always referring to healthcare professionals for further advice. Ms X uses the simulation mode to see the cumulative effect of selected milestones on her treatment choices and refines her entries until they fully match her ideas. She knows that her nursing home physician will be able to use the simulation mode as well, should the case arise. This would allow him to determine the impact of discrete health conditions she had defined before on her goal of care preferences. Ms X went through the d-AD first herself and then once more with her GP, whom she gave access to her d-AD account. He confirms that he discussed the AD with her through checking the respective box in the system. Ms X prints and signs the document which conforms with formal requirements and legal standards for an AD. She deposits a copy with the administration of her senior residency and orders a card with her emergency access code to be put in her wallet.

Although digitally enhanced ADs have considerable potential, a number of issues still need to be solved:

- Extensive user testing needs to ensure that the system is clear and intuitive to users, does not introduce bias or distort patient preferences in any way. Continuous user feedback will be needed to ensure the system continuously improves in a user-driven way. The use of AI (e.g., for predictive purposes) will call for particular caution.
- Given the sensitivity of the data that is collected, processed and shared, any AD app needs to strictly follow national and international data privacy and security standards. For example, the risk of identity theft can be reduced by password protection using two factor authentication. In addition, analyzing access control logs allows for early detection of potentials threats—a process in which artificial intelligence will be extremely helpful. It would be fair to offer users one-time access to generate an AD without any data storage or the option to create an account, where data will be stored on a secure server and can be accessed for easy updates and in case of emergency. In that case clients need to be informed about any further purpose the data may be used for.
- As terminology and legal requirements for ADs as well as other contextual (e.g., cultural) factors differ internationally, country-specific versions would need to be developed if the tool is to be made globally available.
- A digitally enhanced AD app can provide the greatest potential as a part of a digital ecosystem (e.g., an electronic health record) that instigates access to relevant data in the case of a critical health care event.
- Automated reminders need to be sent on a regular basis to check that the ACC still reflects current preferences. This ensures that authorized third-party users (e.g., the treating physician) always have access to up-to-date information about the client's advance care choices.

User trust, acceptance by healthcare providers, and effectiveness in clinical settings will critically depend on how well these points can be addressed.

## Conclusions And Future Perspectives

The Covid-19 pandemic has fueled the digital transformation of healthcare. Although concepts such as the electronic health record or online informed consent have been available for some time (25), implementation has been amazingly slow in many countries. We can expect this to change quickly now, even though a number of issues remain to be addressed regarding reimbursement, liability, care coordination and appropriate research methodologies to assess the impact of new developments on patient-centered care (26, 27). We anticipate digitally enhanced ADs will be part of these developments and believe they can meet the core challenges ADs encounter and that other initiatives have not been able to fully address.

Some limitations of Ads, particularly the principle epistemic limitation of imagining a future health state that has never been experienced, may remain, but technologies such as Virtual Reality (VR) might help improve the situation. Digitally enhanced ADs can be enriched with infographics or stories that provide information about different treatment options. They can be complemented with modules for specific health conditions or populations. Voice editions can remove barriers for citizens with visual impairment or with low literacy. Ideally, digital ADs will be linked with a patient-driven electronic health record and embedded in a patient-oriented ecosystem. Continuous user input and feedback, from patients, proxies and health care professionals, will help the system steadily learn and improve.

Once the concept has been validated including studies of different user groups, settings and interfaces, content could be enriched to include information about various common health conditions such as cancer and dementia. Today it is an open question if the d-AD can reach its aspiration of unfolding a beneficial impact on health care by helping health care professionals understand what their patient would have wanted could he or she talk to them. A well-designed RCT will go some way toward better understanding the potential as well as mechanisms and limitations. Contextual factors, ranging from incentives and disincentives to cultural attitudes toward using digital tools for highly private and important decisions, as well as human-machine interaction will need to be explored with the help of qualitative approaches.

Eventually, the key idea of digitally transforming analog decisions and making them amenable quantification as well as the other features of digitally enhanced decision-support could be applied to health care decisions outside the setting of an advance directive, e.g., choosing the most suitable therapeutic approach in a shared decision-making setting.

We have presented our concept at an early stage in the hope of receiving feedback from an interdisciplinary community of digital health experts and are keen to discover opportunities for collaborations and exchange that will allow us to reach our goal of contributing to a patient-oriented healthcare systems.

## Data Availability Statement

The original contributions presented in the study are included in the article, further inquiries can be directed to the corresponding author.

## Author Contributions

NB-A and AB contributed to conception and design of the study. AB provided the graphs. NB-A wrote the first draft of the manuscript. Both authors contributed to manuscript revision, read, and approved the submitted version.

## Funding

The authors gratefully acknowledge support from the Collegium Helveticum, a transdisciplinary Institute of Advanced Study sponsored jointly by the University of Zurich, the Federal Institute of Technology Zurich and the Zurich University of the Arts.

## Conflict of Interest

The authors declare that the research was conducted in the absence of any commercial or financial relationships that could be construed as a potential conflict of interest.

## Publisher's Note

All claims expressed in this article are solely those of the authors and do not necessarily represent those of their affiliated organizations, or those of the publisher, the editors and the reviewers. Any product that may be evaluated in this article, or claim that may be made by its manufacturer, is not guaranteed or endorsed by the publisher.
